# Machine learning analysis with the comprehensive index of corneal tomographic and biomechanical parameters in detecting pediatric subclinical keratoconus

**DOI:** 10.3389/fbioe.2023.1273500

**Published:** 2023-12-06

**Authors:** Shengwei Ren, Kaili Yang, Liyan Xu, Qi Fan, Yuwei Gu, Chenjiu Pang, Dongqing Zhao

**Affiliations:** Henan Provincial People’s Hospital, Henan Eye Hospital, Henan Eye Institute, People’s Hospital of Zhengzhou University, Henan University People’s Hospital, Zhengzhou, China

**Keywords:** pediatric keratoconus, subclinical keratoconus, corneal tomographic parameters, corneal biomechanical parameters, machine learning, diagnosis ability

## Abstract

**Background:** Keratoconus (KC) occurs at puberty but diagnosis is focused on adults. The early diagnosis of pediatric KC can prevent its progression and improve the quality of life of patients. This study aimed to evaluate the ability of corneal tomographic and biomechanical variables through machine learning analysis to detect subclinical keratoconus (SKC) in a pediatric population.

**Methods:** Fifty-two KC, 52 SKC, and 52 control pediatric eyes matched by age and gender were recruited in a case-control study. The corneal tomographic and biomechanical parameters were measured by professionals. A linear mixed-effects test was used to compare the differences among the three groups and a least significant difference analysis was used to conduct pairwise comparisons. The receiver operating characteristic (ROC) curve and the Delong test were used to evaluate diagnostic ability. Variables were used in a multivariate logistic regression in the machine learning analysis, using a stepwise variable selection to decrease overfitting, and comprehensive indices for detecting pediatric SKC eyes were produced in each step.

**Results:** PE, BAD-D, and TBI had the highest area under the curve (AUC) values in identifying pediatric KC eyes, and the corresponding cutoff values were 12 μm, 2.48, and 0.6, respectively. For discriminating SKC eyes, the highest AUC (95% CI) was found in SP A1 with a value of 0.84 (0.765, 0.915), and BAD-D was the best parameter among the corneal tomographic parameters with an AUC (95% CI) value of 0.817 (0.729, 0.886). Three models were generated in the machine learning analysis, and Model 3 (y = 0.400*PE + 1.982* DA ratio max [2 mm]−0.072 * SP A1−3.245) had the highest AUC (95% CI) value, with 90.4% sensitivity and 76.9% specificity, and the cutoff value providing the best Youden index was 0.19.

**Conclusion:** The criteria of parameters for diagnosing pediatric KC and SKC eyes were inconsistent with the adult population. Combined corneal tomographic and biomechanical parameters could enhance the early diagnosis of young patients and improve the inadequate representation of pediatric KC research.

## 1 Introduction

Keratoconus (KC) is a corneal disorder characterized by an anterior protrusion of the cornea and corneal thinning. (Gomes et al., 2015). It affects all ethnic groups, with the highest prevalence reported in China (0.9%, approximately 12.5 million), India (2.3%, approximately 30 million), and Iran (4% of the rural population, approximately 3.4 million). (Hashemi et al., 2020; Rafat et al., 2022). KC is the leading indication for corneal transplantation, accompanied by serious vision deterioration and irregular astigmatism, and the social and financial burden is remarkable. (Rebenitsch et al., 2011; Rafat et al., 2022).

As a progressive disease, KC typically occurs at puberty and continues to progress until the third or fourth decade of life. (Mukhtar and Ambati, 2018). In addition, several studies have reported that KC is more frequently presented at an advanced stage in young patients than in adults, and pediatric patients with limited cognitive ability are less aware of unilateral visual loss. (Leoni-Mesplie et al., 2012; Ferdi et al., 2019). The persistent underrepresentation of children in KC research has resulted in an inadequate evidence base; therefore, increasing attention to pediatric KC patients is of great value in evaluating the occurrence and development of KC.

Moderate or severe stages of KC are easily diagnosed according to slit lamp findings and topography signs, whereas the detection of abnormal corneas in very early stages or subclinical forms is still a challenge for clinicians. (Mas Tur et al., 2017; Cao et al., 2021; Zhang et al., 2022). Epidemiological studies have revealed that corneal topographic, tomographic, and biomechanical parameters can accurately distinguish a KC or subclinical KC (SKC) eye from a normal eye. (Vinciguerra et al., 2016; Sedaghat et al., 2018; Kataria et al., 2019; Koh et al., 2019). However, the above study mostly focused on adults, who received examinations before refractive surgery mainly to avoid postoperative ectasia. As the clinical manifestation of KC in children is somewhat different from adults, the diagnosis results obtained in adults may not apply to pediatric patients. (Mukhtar and Ambati, 2018; Anitha et al., 2021). However, the diagnosis of pediatric KC is still limited. (Leoni-Mesplie et al., 2012; Wajnsztajn et al., 2021). A retrospective observational study conducted in Egypt reported the eight most useful Pentacam indices in identifying 48 eyes of pediatric KC and SKC. (Hashem et al., 2022). Furthermore, the corneal biomechanics, which are thought to be the initiators of the disease even before notable changes in corneal morphology, were not evaluated in the above study. (Scarcelli et al., 2014; Vinciguerra et al., 2016). A prospective study conducted in Atlanta showed that pediatric KC patients with trisomy 21 had thinner corneas and lower corneal resistance factors than control patients, which were inconsistent with the results in adults. (Neustein and Lenhart, 2022). The above corneal biomechanics were measured by ORA, which has a limited examination area on the cornea and a worse discriminative ability in KC eyes than Corvis ST. (Esporcatte et al., 2023). As an ultra-high speed Scheimpflug camera, Corvis ST can produce various corneal biomechanical parameters and its discriminative ability has been demonstrated to be high in adults. Additionally, studies in pediatric KC patients have been limited. (Vinciguerra et al., 2016; Kataria et al., 2019).

With the advancement of technology, machine learning methods can improve diagnostic ability by incorporating a large amount of data. (Angraal et al., 2020). Several studies have reported the application of machine learning techniques in the field of KC and refractive surgery screening; however, their application has been limited in pediatric KC patients. (Ruiz Hidalgo et al., 2016; Malyugin et al., 2021). Thus, the current study aimed to evaluate the clinical characteristics of KC and SKC eyes in pediatric patients, and explore the diagnostic ability of corneal tomographic and biomechanical parameters in discriminating pediatric SKC eyes, to provide references for the early diagnosis and management of pediatric patients.

## 2 Materials and methods

### 2.1 Study participants

This case-control study enrolled 52 control eyes, 52 SKC eyes, and 52 KC eyes of individuals aged <18 years between January 2019 and January 2022 in the Henan Eye Hospital. KC was included as per previous criteria: (Yang et al., 2022): an asymmetric bowtie pattern with or without skewed axes, a Belin Ambrosio enhanced ectasia total deviation index (BAD-D) value > 2.6 by corneal topography, and a positive slit-lamp sign (localized stromal thinning, Vogt’s striae, Fleischer’s ring, conical protrusion, or anterior stromal scar). The SKC eye in the current study was defined as no clear evidence of KC in one eye, with the contralateral eye meeting the above KC diagnostic criteria. (Ren et al., 2021). The detailed criteria of SKC eye are as follows: without an asymmetric bowtie pattern, a BAD-D value ≤ 2.6 by corneal topography, and no positive slit-lamp sign. Volunteers with a spherical equivalent <8.00 diopters (D), astigmatism <2.00 D, corrected distance visual acuity (CDVA) ≥0.8, and normal corneal topography were recruited in the control group. Eyes with an anterior stromal scar, acute corneal hydrops, soft contact lens wear within the last 2 weeks, rigid contact lens wear within the last 4 weeks, ocular trauma history, ocular surgery history, and other ocular disease history were excluded in the current analysis. Finally, 52 KC eyes, 52 SKC eyes, and 52 control eyes (52 subjects) in pediatric patients matched with age (less than 3 years) and gender were recruited in the analysis.

This study was conducted according to the Declaration of Helsinki guidelines and approved by the Institutional Review Board of the Henan Eye Hospital [ethical approval number: HNEECKY-2019 (5)]. Informed consent was obtained from the legal guardians of pediatric patients.

### 2.2 Examinations

The clinical examinations were conducted when the patients were first referred to the center. All measurements (slit-lamp examination, CDVA in the logarithm of the minimum angle of resolution (logMAR) unit, corneal tomographic, and biomechanical measurements) were obtained by experienced operators between 9:00 and 17:00.

The Pentacam HR (Oculus, Wetzlar, Germany, software number: 1.21r41) is a corneal topography system that uses a rotating high-resolution camera to analyze the anterior segment of the eye. (de Luis Eguileor et al., 2018). The finding with a high-quality factor was recorded for each eye, and the following parameters were analyzed: the central 3.0 mm of the anterior corneal surface in terms of flat keratometry (K1 F), steep keratometry (K2 F), corneal keratometry astigmatism (Ka, the value of K2 minus K1), maximum keratometry (Kmax F), and mean keratometry (Kmean F), the corneal thickness at the pachy apex (ACT), the pupil’s center (PCT), the thinnest point of the cornea (TCT), the thinnest corneal point (front (FE) and posterior elevation values (PE)), and BAD-D values. The Corvis ST (Oculus, Wetzlar, Germany, software number: 1.6b2224) collects parameters during the first applanation, highest concavity, and second applanation phases. (Vinciguerra et al., 2016). The intraocular pressure (IOP), biomechanical corrected intraocular pressure (bIOP), maximum value of the ratio between the deformation amplitude at the apex 1 mm and 2 mm from the central cornea (DA ratio max [1 mm] and DA ratio max [2 mm]), maximum inverse radius, integrated radius, Ambrósio’s relational thickness horizontal (ARTh), stiffness parameter at the first applanation (SP A1), Corvis biomechanical index (CBI), and stress-strain index (SSI) were recorded. In addition, the tomographic and biomechanical index (TBI) was obtained by combining Pentacam HR and Corvis ST measurements.

### 2.3 Analytical Tools and methods

The median and interquartile ranges (P25 and P75) were applied to describe qualitative data. A linear mixed-effects test was used to compare the differences among the three groups and a least significant difference (LSD) analysis was used to conduct pairwise comparisons. The comparisons of corneal biomechanical parameters among different groups have been corrected to adjust for corneal thickness and IOP. (Ren et al., 2021). The receiver operating characteristic (ROC) curve and the Delong test were used to evaluate the diagnostic ability of distinguishing SKC and KC eyes from control eyes. Among that, cutoff (the classification effect is best), sensitivity (the ability of the model to detect patients), specificity (the ability of the model to identify non-patients), Youden index (the sum of sensitivity and specificity minus 1), and area under the ROC curve (AUC, a higher value reflects a better accuracy rate) values and 95% confidence interval (CI) were recorded. (Mandrekar, 2010). The machine learning analysis was conducted through multivariate logistic regression using SPSS 23.0, and the logistic regression was used with forward stepwise selection to choose variables with statistically significant results (*p* < 0.05) in a likelihood ratio test to decrease overfitting. (Angraal et al., 2020). Three steps were generated in the analysis and each step formed a model in the analysis, and the ability of the model to detect pediatric SKC eyes was evaluated through ROC analysis using MedCalc software. *p* < 0.05 (two-tailed) was considered as a statistically significant difference.

## 3 Results

### 3.1 Characteristics of the general parameters

In the present study, the male and female ratio was 42:10 and the median age was 16 years (12.25, 17) and 15.5 years (14, 16.75) for the control and KC patients, respectively (*p* = 0.320). SKC eyes and control eyes had higher CDVA (LogMAR) values than KC eyes (*p* < 0.05), and no significant difference was found in the two groups (*p* = 1.000, Table 1).

**TABLE 1:** Comparisons of general parameters among control, SKC and KC eyes in pediatric subjects.

Parameters	Control (N = 52)	SKC (N = 52)	KC (N = 52)	P*	*P* ^#1^	*P* ^#2^	*P* ^#3^
Gender, n (%)				1.000	-	-	-
Male	42 (80.16)	42 (80.16)	42 (80.16)				
Female	10 (19.23)	10 (19.23)	10 (19.23)				
Age, M (P25, P75)	16.00 (12.25, 17.00)	15.50 (14.00, 16.75)	15.50 (14.00, 16.75)	0.320	-	-	-
CDVA (LogMAR), M (P25, P75)	0 (0, 0)	0 (0, 0)	0.30 (0.15, 0.52)	<0.001	1.000	<0.001	<0.001

*Kruskal–Wallis test; #1 control vs. SKC; #2 control vs. KC; #3 SKC vs. KC.

SKC, subclinical keratoconus; KC, keratoconus; CDVA, corrected distance visual acuity.

### 3.2 Comparisons of the parameters in pediatric control, SKC and KC eyes

**FIGURE 1:**
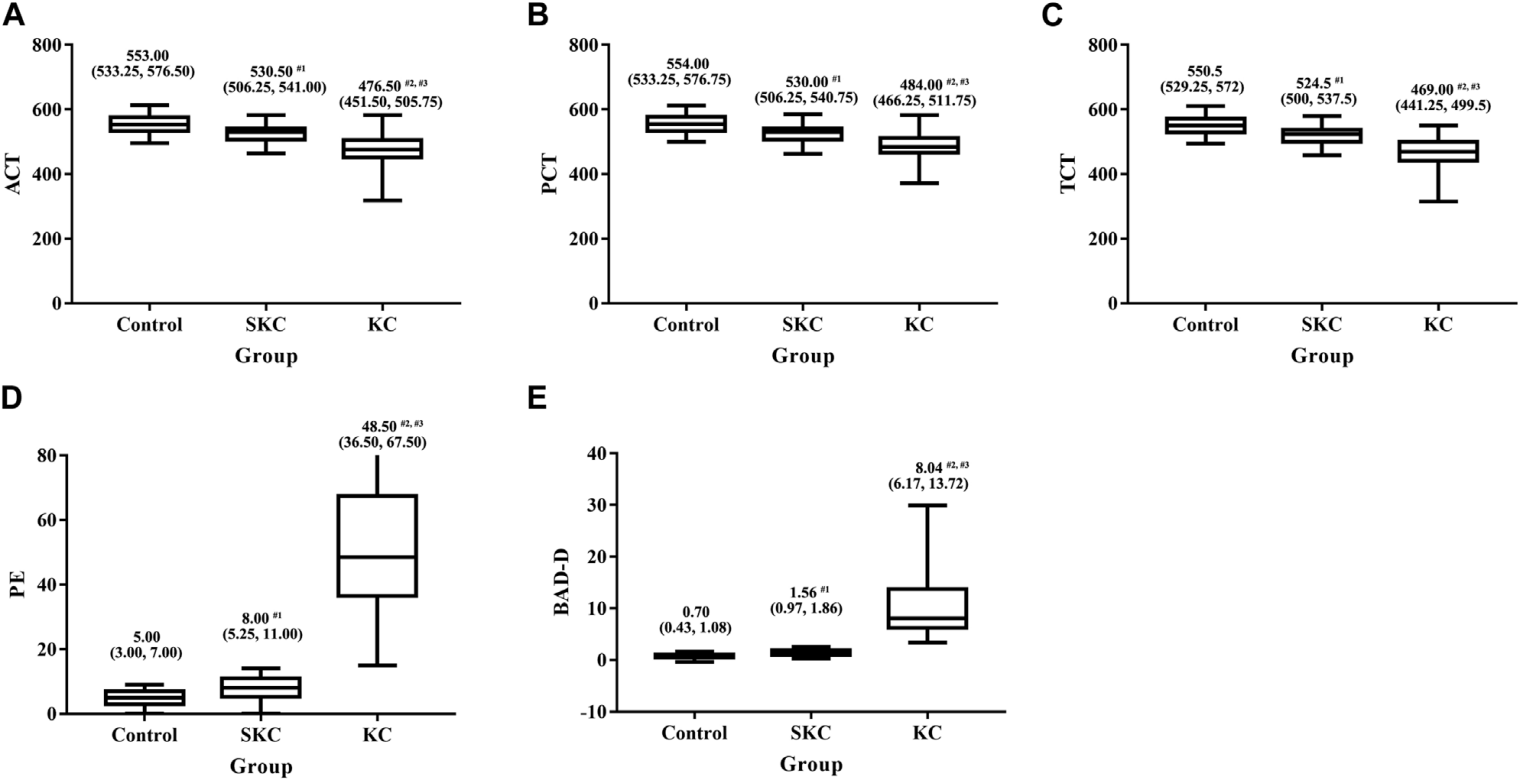
Box plot for significantly different corneal tomographic parameters among control, SKC, and KC eyes in pediatric subjects. **(A)** ACT: the corneal thickness at the pachy apex. **(B)** PCT: the corneal thickness at the pupil’s center. **(C)** TCT: corneal thickness at the thinnest point of the cornea. **(D)** PE: the posterior thinnest corneal point. **(E)** BAD-D: Belin Ambrosio enhanced ectasia total deviation index. Bars represent the median (P25, P75). *p* < 0.001 for all three groups. ^#1^ *p*<0.05 for SKC vs. control; ^#2^ *p*<0.05 for KC vs. control; ^#3^ *p*<0.05 for KC vs. SKC.

**FIGURE 2:**
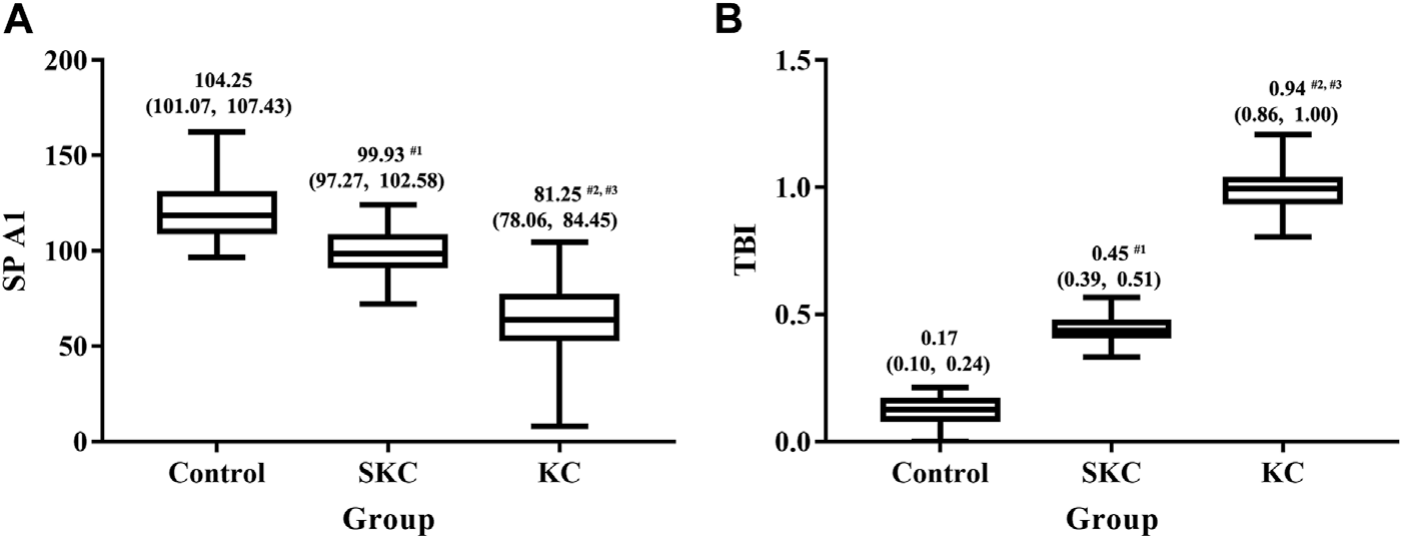
Box plot for SPA1 and TBI among control, SKC, and KC eyes in pediatric subjects. **(A)** SP A1: stiffness parameter at the first applanation. **(B)** TBI: tomographic and biomechanical index. Bars represent the median (P25, P75). *p* < 0.001 for all three groups. ^#1^ *p*<0.05 for SKC vs. control; ^#2^ *p*<0.05 for KC vs. control; ^#3^ *p*<0.05 for KC vs. SKC.

Significant differences in corneal tomographic and corneal biomechanical parameters were found among control, SKC and KC eyes in pediatric subjects (*p* < 0.05, Supplementary Tables S1, S2). Pairwise analyses found that SKC eyes of pediatric patients had lower ACT, PCT, and TCT values and higher PE and BAD-D values than control eyes (all *p* < 0.05, Figure 1). Furthermore, KC and SKC eyes had higher TBI values and lower SP A1 values than control eyes after adjusting for IOP and corneal thickness (*p* < 0.05, Figure 2).

### 3.3 Diagnostic ability of corneal tomographic and biomechanical parameters

**FIGURE 3:**
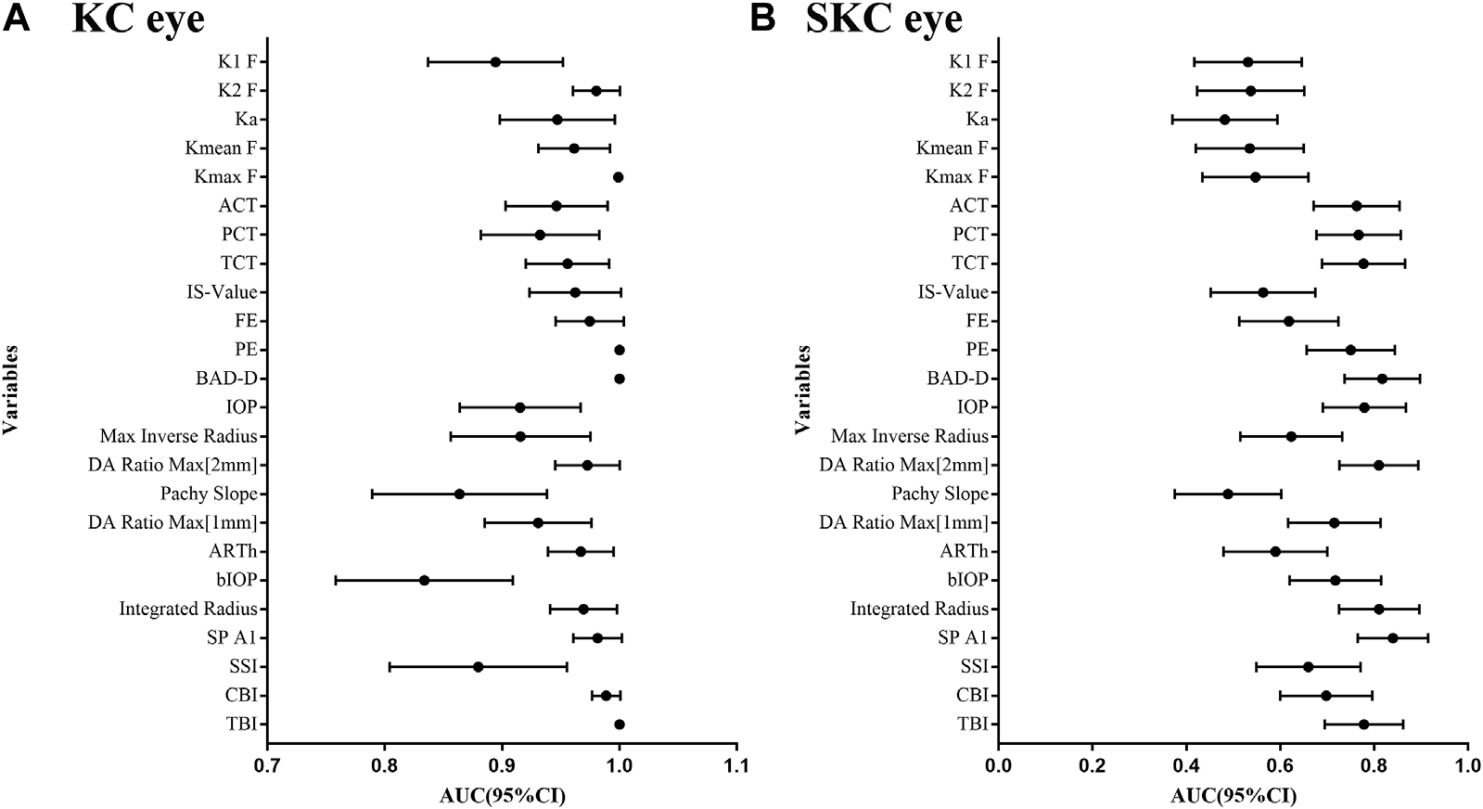
AUC values for corneal tomographic and biomechanical variables in diagnosing pediatric SKC and KC eyes. **(A)** Pediatric KC eyes. **(B)** pediatric SKC eyes.

Supplementary Table S3 shows the ability of corneal parameters to identify pediatric KC eyes. For corneal tomographic parameters, PE and BAD-D had the highest diagnostic values (AUC = 1), and the corresponding cutoff values were 12 μm and 2.48, respectively. For corneal biomechanical parameters, CBI and SP A1 were excellent, with AUC values of 0.990 (cutoff value: 0.53) and 0.985 (cutoff value: 96.69), respectively. Combined parameters of TBI also had the highest diagnostic values (AUC = 1) at a cutoff value of 0.60 (Figure 3A).

Additionally, the AUC values of corneal parameters in identifying pediatric SKC eyes are shown in Figure 3B. BAD-D was the best parameter among the corneal tomographic parameters, with an AUC (95% CI) value of 0.817 (0.729, 0.886), and the cutoff value providing the best Youden index was 1.14. For corneal biomechanical parameters, the highest AUC (95% CI) was found in SP A1 with 0.840 (0.765, 0.915), and the cutoff value providing the best Youden index was 108.33 mmHg/mm. This was followed by integrated radius and DA ratio max [2 mm], which had AUC values of 0.811 (cutoff value: 8.89) and 0.810 (the cutoff value: 4.37), respectively. The AUC (95% CI) of TBI was 0.784 (0.692, 0.859) and the cutoff value providing the best Youden index was 0.35. The diagnostic abilities of other parameters are shown in Supplementary Table S4.

### 3.4 Diagnostic ability of comprehensive parameters to detect SKC eye

**FIGURE 4:**
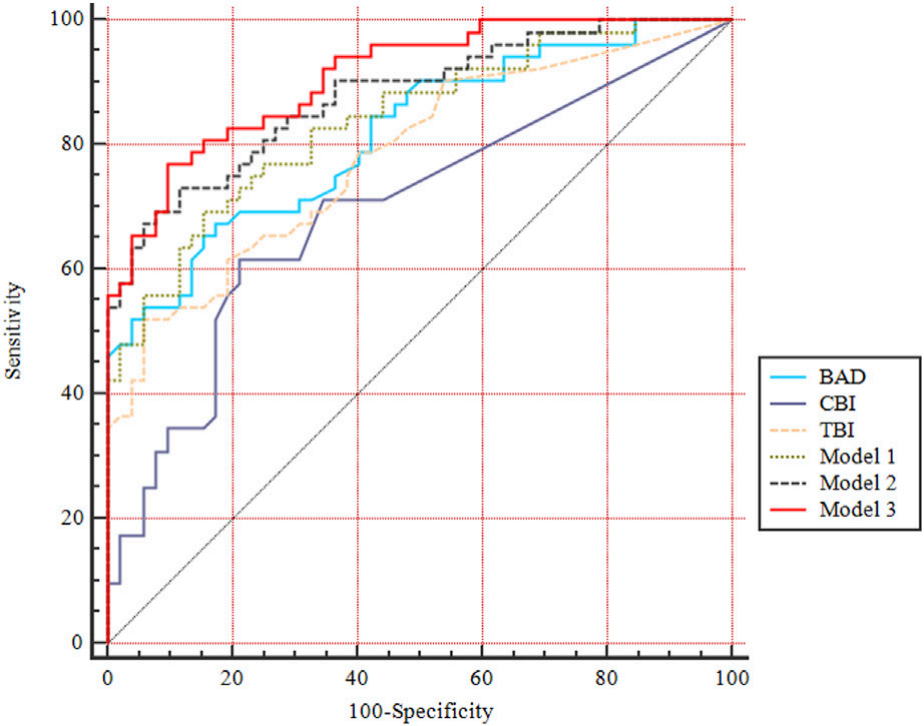
ROC curves of comprehensive indices for diagnosing pediatric SKC eyes. BAD-D, Belin Ambrosio enhanced ectasia total deviation index; CBI, Corvis biomechanical index; TBI, tomographic and biomechanical index. Model 1, y =—0.105*SP A1+11.405; Model 2, y = 0.314*PE—0.097*SP A1+8.590; Model 3, y = 0.400*PE +1.982* DA ratio max [2 mm]—0.072*SP A1–3.245.

Three models were separately generated from each step of the multivariate logistic regression in the machine learning analysis (Supplementary Table S5). The ability of comprehensive indices in detecting pediatric SKC eyes is shown in Table 2 and Figure 4. Model 3 (y = 0.400*PE +1.982* DA ratio max [2 mm]−0.072*SP A1−3.245) had the highest AUC (95% CI) value, with a sensitivity of 90.4% and a specificity of 76.9%, and the cutoff value providing the best Youden index was 0.19. Further pairwise comparisons indicated that the AUC value of Model 3 was higher than BAD, CBI, TBI, and Model 1(*p*<0.05), and no significant difference was found compared with Model 2 (y = 0.314*PE−0.097*SP A1+8.590, *p* > 0.05, Supplementary Table S6).

**TABLE 2:** Ability of comprehensive indices in distinguishing pediatric SKC eyes from control eyes.

Parameters	Cutoff	Specificity	Sensitivity	Youden index	AUC (95% CI)
BAD	1.14	0.827	0.673	0.500	0.817 (0.729, 0.886)
CBI	0.02	0.789	0.615	0.404	0.698 (0.600, 0.784)
TBI	0.35	0.942	0.519	0.462	0.784 (0.692, 0.859)
Model 1	>0.01	0.846	0.692	0.539	0.840 (0.755, 0.904)
Model 2	>0.53	0.923	0.692	0.615	0.879 (0.801, 0.935)
Model 3	>0.19	0.904	0.769	0.673	0.909 (0.837, 0.957)

AUC, area of receiver operating characteristic curve; CI, confidence interval; BAD-D, belin ambrosio enhanced ectasia total deviation index; CBI, corvis biomechanical index; TBI, tomographic and biomechanical index.

Model 1 y = −0.105*SP A1+11.405.

Model 2 y = 0.314*PE−0.097*SP A1+8.590.

Model 3 y = 0.400*PE +1.982* DA ratio max [2 mm]−0.072*SP A1−3.245.

## 4 Discussion

As KC in young patients is reported to be more likely to progress than in adults, the sensitivity index and cutoff values of parameters in pediatric patients is different than in adult patients. (Hashem et al., 2022; Neustein and Lenhart, 2022). The machine learning analysis, analyzing a large number of variables, found that the comprehensive index, containing PE, DA ratio max [2 mm], and SP A1, improved the diagnostic capability to differentiate pediatric SKC eyes.

Pentacam is one of the most common techniques in clinical application, as the uniqueness of this device is that it includes tomographic data, topometric data, pachymetry, elevation data, and combined data. (Motlagh et al., 2019). In the present study, BAD-D and PE were highly capable of differentiating pediatric KC and SKC eyes, which was consistent with previous findings in adult patients with similar criteria. BAD-D is a multivariate index that considers several separate indices, and its AUC value in diagnosing adult KC was 0.990, and the cutoff value providing the best Youden index was 2.38 in the study by Hashemi et al., 2016. In the study by Ambrosio et al. (2013) the AUC value in detecting adult SKC eyes was 0.975 and the cutoff value providing the best Youden index was 1.22. PE represents the maximum evaluation of the corneal posterior in a zone above the standardized reference shape, and this variable is widely regarded as the earliest indicator of ecstatic change. (Saad and Gatinel, 2010; Motlagh et al., 2019). The AUC value of PE in diagnosing adult KC was 0.991, and the cutoff value providing the best Youden index was 12 μm in the study by Ambrosio et al., 2013. The AUC value in detecting SKC was 0.882 and the cutoff value providing the best Youden index was 7.5 μm in the study by Du XL et al. (Du et al., 2015), which were both higher than in pediatric patients at the same cutoff values in the current study. In general, the diagnostic efficacy of corneal tomographic variables in pediatric patients was higher than in adult patients at a similar cutoff value, which suggested that caution is warranted when using these parameters in different age groups. (Hashem et al., 2022).

The diagnostic criteria of corneal tomographic parameters in pediatric KC are still limited. (Motlagh et al., 2019; Hashem et al., 2022). A prospective study reported that pediatric KC with trisomy 21 has a thinner cornea, with a lower TCT and higher values of Kmax and K2 than normal children, which was consistent with the current results. (Neustein and Lenhart, 2022). In addition, the current study found that the CCT and K1 were significantly different between KC and the control, whereas no significant difference was found in the study by Neustein and Lenhart, 2022 These discrepancies may be attributed to the sample size, ethnicity, and characters of the subjects. In addition, Hashem et al., 2022 reported that relational thickness and other OCULUS values analyzed for the 8-mm zone (CAIRO 8) were the most effective for KC detection in pediatric eyes, which is inconsistent with the present study. On one hand, the parameters included in the analyses were not completely consistent, e.g., BAD-D, which has been widely demonstrated to be one of the best indices for identifying KC and SKC eyes, was not mentioned in the study by Hashem AO et al., and CAIRO 8 was not analyzed in the present study. (Hashemi et al., 2016; Hashem et al., 2022). On the other hand, Hashem et al., 2022 included 40 KC and 8 SKC eyes as a whole case group, despite the inconsistency between SKC and KC eyes, and the KC and SKC eyes were analyzed separately in the current study. The limited sample of pediatric SKC eyes would result in a lack of research evidence of the diagnosis criteria of pediatric SKC eyes. In addition, our previous study reported that pediatric unilateral patients account for 7.82% of all KC patients (86/1,100), (Yang et al., 2022), which might be related to the fact that the minimum age approved for corneal refractive surgery is 18 years and corneal tomography and biomechanics are not commonly examined in children. (Ortega-Usobiaga et al., 2022). Thus, more attention should be paid to children with sight loss, and deep studies are needed to further verify the ability of corneal topographic parameters in pediatric patients.

With the increasing understanding that the changes in corneal biomechanics precede tomography, more attention has been focused on corneal biomechanics in the early diagnosis of KC. (Vinciguerra et al., 2016; Moshirfar et al., 2019; Herber et al., 2022). Corvis ST has been increasingly evaluated in the detection of early adult KC. Evidence of Corvis ST parameters in pediatric KC and SKC is still lacking. (Li et al., 2023). SP A1 is a novel stiffness parameter that is defined as resultant pressure divided by deflection at the first applanation, and the KC eye tends to have a smaller value that represents a weaker cornea. (Vinciguerra et al., 2016; Yang et al., 2020). The present study found that SP A1 in pediatric SKC eyes was lower than that in control eyes after adjusting for IOP and corneal thickness, which indicated that the cornea of the pediatric SKC eye weakened. In addition, the abilities of CBI, SP A1, and DA ratio max (2 mm) in diagnosing pediatric KC eyes were high, which was consistent with previous studies of adult KC. (Kataria et al., 2019; Moshirfar et al., 2019; Ren et al., 2021). DA ratio max [2 mm] is the deformation amplitude measured 2 mm from the corneal center, and a higher value represents a softer cornea or one that is less resistant to cornea deformation. (Yang et al., 2019). CBI, a parameter of the logistic regression analysis, was useful in discriminating KC when the value was higher than 0.5. (Vinciguerra et al., 2016). The AUCs of SP A1 and integrated radius in the present study were higher than those in a previous study of adult SKC patients, which further indicated that the corneal tests for the evaluation of adults might not be reliable at identifying pediatric patients. (Neustein and Lenhart, 2022). Integrated radius is the integrated area under the curve of the inverse radius, and the KC eye has a higher value than the normal eye. (Ren et al., 2021). In addition, the accuracy of CBI in detecting adult SKC is excellent and relatively higher than that in pediatric SKC eyes in the present study. (Kataria et al., 2019; Ren et al., 2021). The results suggested that the criteria and cutoff values of corneal biomechanics in adult KC and SKC may not suitable for pediatric patients, which should be paid more attention to in clinical application and more comparative studies are needed in the future.

TBI is combined data that are synergistically integrated and has been widely demonstrated to be useful in detecting corneal ectasia. (Koh et al., 2019; Moshirfar et al., 2019). The present study found that TBI was the best variable in detecting pediatric KC eyes. As for diagnosing pediatric SKC eyes, the AUC of TBI in the present study was lower than that in adult SKC eyes. (Ambrosio et al., 2017; Koc et al., 2019). In addition, the ability of TBI was weaker than the combined model obtained through machine analysis. The comprehensive index of PE, DA ratio max [2 mm], and SP A1 could effectively discriminate pediatric SKC eyes from control eyes, providing a reference for exploring the early detection of pediatric patients. The current diagnosis criteria included corneal tomographic and corneal biomechanical parameters, and the accuracy of diagnosing pediatric SKC eye was significantly higher than CBI, DA ratio max [2 mm], SP A1, PE, and integrated radius. Several dynamic corneal response (DCR) parameters of insufficient diagnostic ability could improve the diagnostic ability but cannot currently be considered standalone parameters for screening purposes. (Ali et al., 2014). The accuracy of other DCR variables in detecting SKC eyes was limited reported, and the combined diagnostic mechanism of these parameters needs further evaluation in engineering mechanics.

At present, insufficient attention is being paid to vision loss in children, which has a higher progressive rate than adult patients. Early diagnosis of pediatric KC and further effective early treatments can prevent the need for corneal transplantation and improve the quality of life of patients. (Neustein and Lenhart, 2022). Several limitations must be acknowledged. First, the sample of pediatric SKC eyes included in current study was not large enough, although the population was drawn from a relatively large KC population. Second, the study was conducted in a single center, with strict grouping criteria and matched with age and gender to control bias. Therefore, the results still need to be further validated at other centers. Third, the machine learning methods improved the prediction ability of pediatric SKC eyes, but the validation for the machine learning analysis result was lacking. Including a large number of subjects in the validation analysis is necessary and the study is ongoing and continuing to collect unilateral pediatric KC patients for further validation. Thus, a multicenter pediatric study with a large sample size is needed in the future. Finally, the present machine learning method assumed the input variables and outcomes were probably linear, and non-linear was not considered. Therefore, the possibility of assessing alternative classifiers based on algorithms other than a linear model should be conducted in the future.

In conclusion, the ability of corneal tomographic and biomechanical variables and their cutoff values in detecting pediatric KC and SKC eyes was inconsistent in adult patients, and combined parameters had better discriminative efficacy than single instrument variables in detecting SKC eyes. Relative findings will provide sensitive indices for the follow-up management of unilateral pediatric KC and a reference for the early diagnosis of children suspected to have KC.

## Data Availability

The original contributions presented in the study are included in the article/Supplementary Material, further inquiries can be directed to the corresponding author.

## Abbreviations

KC, Keratoconus; SKC, Subclinical keratoconus; ROC, Receiver operating characteristic; BAD-D, Belin Ambrosio enhanced ectasia total deviation index; CDVA, Corrected distance visual acuity; K1 F, Flat keratometry; K2 F, Steep keratometry; Ka, Corneal keratometric astigmatism; Kmax F, The maximum keratometry; Kmean F, Mean keratometry; ACT, Corneal thickness at the pachy apex; PCT, Corneal thickness at the pupil’s center; TCT, Corneal thickness at the thinnest point of the cornea; FE, Anterior evaluation; PE, Posterior elevation; IOP, Intraocular pressure; bIOP, Biomechanical corrected intraocular pressure; ARTh, Ambrósio’s relational thickness horizontal; SP A1, Stiffness parameter at the first applanation; CBI, Corvis biomechanical index; SSI, Stress-strain index; TBI, Tomographic and biomechanical index; LSD, Least significant difference; AUC, Area under the receiver operating characteristic curve; CI, Confidence interval.

## References

[B1] Ali N. Q. Patel D. V. McGhee C. N. (2014). Biomechanical responses of healthy and keratoconic corneas measured using a noncontact scheimpflug-based tonometer. Investigative Ophthalmol. Vis. Sci. 55, 3651–3659. 10.1167/iovs.13-13715 24833745

[B2] Ambrosio R. Jr. Lopes B. T. Faria-Correia F. Salomão M. Q. Bühren J. Roberts C. J. (2017). Integration of scheimpflug-based corneal tomography and biomechanical assessments for enhancing ectasia detection. J. Refract. Surg. (Thorofare, N. J. 1995) 33, 434–443. 10.3928/1081597x-20170426-02 28681902

[B3] Ambrosio R. Jr. Valbon B. F. Faria-Correia F. Ramos I. Luz A. (2013). Scheimpflug imaging for laser refractive surgery. Curr. Opin. Ophthalmol. 24, 310–320. 10.1097/icu.0b013e3283622a94 23680761

[B4] Angraal S. Mortazavi B. J. Gupta A. Khera R. Ahmad T. Desai N. R. (2020). Machine learning prediction of mortality and hospitalization in heart failure with preserved ejection fraction. JACC Heart Fail. 8, 12–21. 10.1016/j.jchf.2019.06.013 31606361

[B5] Anitha V. Vanathi M. Raghavan A. Rajaraman R. Ravindran M. Tandon R. (2021). Pediatric keratoconus - current perspectives and clinical challenges. Indian J. Ophthalmol. 69, 214–225. 10.4103/ijo.ijo_1263_20 33463562 PMC7933850

[B6] Cao K. Verspoor K. Chan E. Daniell M. Sahebjada S. Baird P. N. (2021). Machine learning with a reduced dimensionality representation of comprehensive Pentacam tomography parameters to identify subclinical keratoconus. Comput. Biol. Med. 138, 104884. 10.1016/j.compbiomed.2021.104884 34607273

[B7] de Luis Eguileor B. Escudero Argaluza J. Pijoan Zubizarreta J. I. Santamaria Carro A. Etxebarria Ecenarro J. (2018). Evaluation of the reliability and repeatability of scheimpflug system measurement in keratoconus. Cornea 37, 177–181. 10.1097/ico.0000000000001373 28957978

[B8] Du X. L. Chen M. Xie L. X. (2015). Correlation of basic indicators with stages of keratoconus assessed by Pentacam tomography. Int. J. Ophthalmol. 8, 1136–1140. 10.3980/j.issn.2222-3959.2015.06.10 26682161 PMC4651877

[B9] Esporcatte L. P. G. Salomao M. Q. Lopes B. T. Sena N. Ferreira É. Filho J. B. R. F. (2023). Biomechanics in keratoconus diagnosis. Curr. eye Res. 48, 130–136. 10.1080/02713683.2022.2041042 35184637

[B10] Ferdi A. C. Nguyen V. Gore D. M. Allan B. D. Rozema J. J. Watson S. L. (2019). Keratoconus natural progression: a systematic review and meta-analysis of 11 529 eyes. Ophthalmology 126, 935–945. 10.1016/j.ophtha.2019.02.029 30858022

[B11] Gomes J. A. Tan D. Rapuano C. J. Belin M. W. Ambrósio R. Guell J. L. (2015). Global consensus on keratoconus and ectatic diseases. Cornea 34, 359–369. 10.1097/ico.0000000000000408 25738235

[B12] Hashem A. O. Aziz B. F. Wahba S. S. Roshdy M. M. Elawamry A. I. (2022). Diagnostic accuracy of different keratoconus detection indices of pentacam in paediatric eyes. London, England: Eye. 10.1038/s41433-022-02070-x PMC10102329 35505110

[B13] Hashemi H. Beiranvand A. Yekta A. Maleki A. Yazdani N. Khabazkhoob M. (2016). Pentacam top indices for diagnosing subclinical and definite keratoconus. J. Curr. Ophthalmol. 28, 21–26. 10.1016/j.joco.2016.01.009 27239598 PMC4881219

[B14] Hashemi H. Heydarian S. Hooshmand E. Saatchi M. Yekta A. Aghamirsalim M. (2020). The prevalence and risk factors for keratoconus: a systematic review and meta-analysis. Cornea 39, 263–270. 10.1097/ico.0000000000002150 31498247

[B15] Herber R. Hasanli A. Lenk J. Vinciguerra R. Terai N. Pillunat L. E. (2022). Evaluation of corneal biomechanical indices in distinguishing between normal, very asymmetric, and bilateral keratoconic eyes. J. Refract. Surg. (Thorofare, N. J. 1995) 38, 364–372. 10.3928/1081597x-20220601-01 35686712

[B16] Kataria P. Padmanabhan P. Gopalakrishnan A. Padmanaban V. Mahadik S. Ambrósio R. Jr. (2019). Accuracy of Scheimpflug-derived corneal biomechanical and tomographic indices for detecting subclinical and mild keratectasia in a South Asian population. J. cataract Refract. Surg. 45, 328–336. 10.1016/j.jcrs.2018.10.030 30527442

[B17] Koc M. Aydemir E. Tekin K. Inanc M. Kosekahya P. Kiziltoprak H. (2019). Biomechanical analysis of subclinical keratoconus with normal topographic, topometric, and tomographic findings. J. Refract. Surg. (Thorofare, N. J. 1995) 35, 247–252. 10.3928/1081597x-20190226-01 30984982

[B18] Koh S. Ambrósio R. Jr. Inoue R. Maeda N. Miki A. Nishida K. (2019). Detection of subclinical corneal ectasia using corneal tomographic and biomechanical assessments in a Japanese population. J. Refract. Surg. (Thorofare, N. J. 1995) 35, 383–390. 10.3928/1081597x-20190417-01 31185104

[B19] Leoni-Mesplie S. Mortemousque B. Touboul D. Malet F. Praud D. Mesplié N. (2012). Scalability and severity of keratoconus in children. Am. J. Ophthalmol. 154, 56–62.e1. 10.1016/j.ajo.2012.01.025 22534107

[B20] Li X. Luo S. Wang Z. Miao Y. Zhu M. Zheng X. (2023). Dynamic topography analysis of the cornea and its application to the diagnosis of keratoconus. Comput. Biol. Med. 158, 106800. 10.1016/j.compbiomed.2023.106800 36966554

[B21] Malyugin B. Sakhnov S. Izmailova S. (2021). Keratoconus diagnostic and treatment algorithms based on machine-learning methods, 11. Basel, Switzerland: Diagnostics. 10.3390/diagnostics11101933 PMC8535111 34679631

[B22] Mandrekar J. N. (2010). Receiver operating characteristic curve in diagnostic test assessment. J. Thorac. Oncol. official Publ. Int. Assoc. Study Lung Cancer 5, 1315–1316. 10.1097/jto.0b013e3181ec173d 20736804

[B23] Mas Tur V. MacGregor C. Jayaswal R. O'Brart D. Maycock N. (2017). A review of keratoconus: diagnosis, pathophysiology, and genetics. Surv. Ophthalmol. 62, 770–783. 10.1016/j.survophthal.2017.06.009 28688894

[B24] Moshirfar M. Motlagh M. N. Murri M. S. Momeni-Moghaddam H. Ronquillo Y. C. Hoopes P. C. (2019). Advances in biomechanical parameters for screening of refractive surgery candidates: a review of the literature, Part III. Med. hypothesis, Discov. innovation Ophthalmol. J. 8, 219–240. PMC6778467 31598522

[B25] Motlagh M. N. Moshirfar M. Murri M. S. Skanchy D. F. Momeni-Moghaddam H. Ronquillo Y. C. (2019). Pentacam® corneal tomography for screening of refractive surgery candidates: a review of the literature, Part I. Med. hypothesis, Discov. innovation Ophthalmol. J. 8, 177–203. PMC6778463 31598520

[B26] Mukhtar S. Ambati B. K. (2018). Pediatric keratoconus: a review of the literature. Int. Ophthalmol. 38, 2257–2266. 10.1007/s10792-017-0699-8 28852910 PMC5856649

[B27] Neustein R. F. Lenhart P. D. (2022). Detecting keratoconus: feasibility and findings in three pediatric risk groups. J. Pediatr. Ophthalmol. strabismus 59, 94–101. 10.3928/01913913-20210802-01 34928766

[B28] Ortega-Usobiaga J. Rocha-de-Lossada C. Llovet-Rausell A. Llovet-Osuna F. (2022). Update on contraindications in laser corneal refractive surgery. Arch. Soc. Esp. Oftalmol. Engl. Ed. 98, 105–111. 10.1016/j.oftale.2022.07.003 36114139

[B29] Rafat M. Jabbarvand M. Sharma N. Xeroudaki M. Tabe S. Omrani R. (2022). Bioengineered corneal tissue for minimally invasive vision restoration in advanced keratoconus in two clinical cohorts. Nat. Biotechnol. 41, 70–81. 10.1038/s41587-022-01408-w 35953672 PMC9849136

[B30] Rebenitsch R. L. Kymes S. M. Walline J. J. Gordon M. O. (2011). The lifetime economic burden of keratoconus: a decision analysis using a markov model. Am. J. Ophthalmol. 151, 768–773.e2. 10.1016/j.ajo.2010.10.034 21310384 PMC4714341

[B31] Ren S. Xu L. Fan Q. Gu Y. Yang K. (2021). Accuracy of new Corvis ST parameters for detecting subclinical and clinical keratoconus eyes in a Chinese population. Sci. Rep. 11, 4962. 10.1038/s41598-021-84370-y 33654120 PMC7925657

[B32] Ruiz Hidalgo I. Rodriguez P. Rozema J. J. Ní Dhubhghaill S. Zakaria N. Tassignon M. J. (2016). Evaluation of a machine-learning classifier for keratoconus detection based on scheimpflug tomography. Cornea 35, 827–832. 10.1097/ico.0000000000000834 27055215

[B33] Saad A. Gatinel D. (2010). Topographic and tomographic properties of forme fruste keratoconus corneas. Investigative Ophthalmol. Vis. Sci. 51, 5546–5555. 10.1167/iovs.10-5369 20554609

[B34] Scarcelli G. Besner S. Pineda R. Yun S. H. (2014). Biomechanical characterization of keratoconus corneas *ex vivo* with Brillouin microscopy. Investigative Ophthalmol. Vis. Sci. 55, 4490–4495. 10.1167/iovs.14-14450 PMC4109405 24938517

[B35] Sedaghat M. R. Momeni-Moghaddam H. Ambrosio R. Jr. Heidari H. R. Maddah N. Danesh Z. (2018). Diagnostic ability of corneal shape and biomechanical parameters for detecting frank keratoconus. J. Ophthalmol. 37, 1025–1034. 10.1097/ico.0000000000001639 29847493

[B36] Vinciguerra R. Ambrosio R. Jr. Elsheikh A. Roberts C. J. Lopes B. Morenghi E. (2016). Detection of keratoconus with a new biomechanical index. J. Refract. Surg. (Thorofare, N. J. 1995) 32, 803–810. 10.3928/1081597x-20160629-01 27930790

[B37] Wajnsztajn D. Hopkinson C. L. Larkin D. F. P. (2021). Keratoplasty for keratoconus in young patients: demographics, clinical features, and post-transplant outcomes. Eur. J. Ophthalmol. 226, 68–75. 10.1016/j.ajo.2021.02.003 33577788

[B38] Yang K. Gu Y. Xu L. Fan Q. Zhu M. Wang Q. (2022). Distribution of pediatric keratoconus by different age and gender groups. Front. Pediatr. 10, 937246. 10.3389/fped.2022.937246 35923788 PMC9339668

[B39] Yang K. Xu L. Fan Q. Ren S. (2020). Association between corneal stiffness parameter at the first applanation and keratoconus severity. J. Ophthalmol. 2020, 1–8. 10.1155/2020/6667507 PMC7726963 33343935

[B40] Yang K. Xu L. Fan Q. Zhao D. Ren S. (2019). Repeatability and comparison of new Corvis ST parameters in normal and keratoconus eyes. Sci. Rep. 9, 15379. 10.1038/s41598-019-51502-4 31653884 PMC6814725

[B41] Zhang H. Zhang X. Hua L. Li L. Tian L. (2022). An exploratory analysis of forme fruste keratoconus sensitivity diagnostic parameters. Int. Ophthalmol. 42, 2473–2481. 10.1007/s10792-022-02246-0 35247116

