# Supplementary material for: Machine learning analysis with the comprehensive index of corneal tomographic and biomechanical parameters in detecting pediatric subclinical keratoconus
Source: Front Bioeng Biotechnol. 2023 Dec 6;11:1273500. doi: 10.3389/fbioe.2023.1273500 (PMC10730932; doi:10.3389/fbioe.2023.1273500)
Supplement: Supplementary file 1 [file Table1.doc]

Supplementary Material

# Machine learning analysis with the comprehensive index of corneal tomographic and biomechanical parameters in detecting pediatric subclinical keratoconus

Shengwei Ren^1^, Kaili Yang^*1^, Liyan Xu^1^, Qi Fan^1^, Yuwei Gu^1^, Chenjiu Pang^1^, Dongqing Zhao^1^

^1^ Henan Provincial People’s Hospital, Henan Eye Hospital, Henan Eye Institute, People's Hospital of Zhengzhou University, Henan University People's Hospital, Zhengzhou, 450003, China

*** Correspondence:**Kaili Yang

E-mail: kelly1992abc@163.com

# Supplementary Tables

**Supplemental Table 1** Comparisons of corneal tomographic parameters among pediatric control, SKC and KC eyes

**Supplemental Table 2** Comparisons of corneal biomechanical parameters among pediatric control, SKC and KC eyes

**Supplemental Table 3** Ability of corneal tomographic and biomechanical parameters in identifying pediatric KC eyes

**Supplemental Table 4** Ability of corneal tomographic and biomechanical parameters in identifying pediatric SKC eyes

**Supplemental Table 5** Logistic regression for predicting pediatric SKC eye

**Supplemental Table 6** Delong test results for pairwise comparison of the AUCs in predicting pediatric SKC eye

**Supplemental Table 1 Comparisons of corneal tomographic among pediatric control, SKC and KC eyes**

| **Parameters, M (P25, P75)** | **Control(N=52)** | **SKC(N=52)** | **KC(N=52)** | ***P**** | ***P*^#1^** | ***P*^#2^** | ***P*^#3^** |
| --- | --- | --- | --- | --- | --- | --- | --- |
| K1 F(D) | 41.75(40.85, 43.10) | 42.15(41.50, 42.78) | 45.35(43.40, 49.73) | <0.001 | 1.000 | <0.001 | <0.001 |
| K2 F(D) | 43.00(42.03, 44.75) | 43.55(42.50, 44.30) | 50.70(47.40, 54.83) | <0.001 | 1.000 | <0.001 | <0.001 |
| Ka(D) | 1.20(0.80, 1.68) | 1.20(0.80, 1.60) | 4.50(2.93, 5.95) | <0.001 | 1.000 | <0.001 | <0.001 |
| Kmean F(D) | 42.45(41.50, 43.88) | 42.85(42.10, 43.58) | 48.00(45.40, 52.65) | <0.001 | 1.000 | <0.001 | <0.001 |
| Kmax F(D) | 43.57(42.66, 45.28) | 44.25(42.89, 45.03) | 56.64(51.74, 64.50) | <0.001 | 1.000 | <0.001 | <0.001 |
| ACT(μm) | 553.00(533.25, 576.50) | 530.50(506.25, 541.00) | 476.50(451.50, 505.75) | <0.001 | 0.001 | <0.001 | <0.001 |
| PCT(μm) | 554.00(533.25, 576.75) | 530.00(506.25, 540.75) | 484.00(466.25, 511.75) | <0.001 | <0.001 | <0.001 | <0.001 |
| TCT(μm) | 550.50(529.25, 572.00) | 524.50(500.00, 537.50) | 469.00(441.25, 499.50) | <0.001 | <0.001 | <0.001 | <0.001 |
| IS-Value | 0.07(-0.31, 0.37) | 0.14(-0.27, 0.58) | 3.66(2.55, 6.09) | <0.001 | 1.000 | <0.001 | <0.001 |
| FE(μm) | 2.00(1.00, 3.00) | 2.50(2.00, 4.00) | 20.00(13.25, 37.75) | <0.001 | 0.486 | <0.001 | <0.001 |
| PE(μm) | 5.00(3.00, 7.00) | 8.00(5.25, 11.00) | 48.50(36.50, 67.50) | <0.001 | 0.010 | <0.001 | <0.001 |
| BAD-D | 0.7(0.43, 1.08) | 1.56(0.97, 1.86) | 8.04(6.17, 13.72) | <0.001 | 0.001 | <0.001 | <0.001 |

*Linear mixed effects of three groups, ^#1^ control vs SKC, ^#2^ control vs KC, ^#3^ SKC vs KC

SKC: subclinical keratoconus; KC: keratoconus; K1 F: front flat keratometry; K2 F: front steep keratometry, Ka: keratometric astigmatism; Kmax F: the front maximum keratometry; Kmean F: front mean keratometry; ACT: the corneal thickness at the pachy apex; PCT: the corneal thickness at the pupil's center; TCT: corneal thickness at the thinnest point of the cornea; FE: the front thinnest corneal point; PE: the posterior thinnest corneal point; BAD-D: Belin Ambrosio enhanced ectasia total deviation index.

**Supplemental Table 2 Comparisons of corneal biomechanical parameters among pediatric control, SKC and KC eyes**

| **Parameters, M (P25, P75)** | **Control(N=52)** | **SKC(N=52)** | **KC(N=52)** | ***P**** | ***P*^#1^** | ***P*^#2^** | ***P*^#3^** |
| --- | --- | --- | --- | --- | --- | --- | --- |
| **Crude model** |  |  |  |  |  |  |  |
| IOP(mmHg) | 17.00(15.50, 18.00) | 15.00(13.13, 16.00) | 12.75(11.00, 14.00) | <0.001 | <0.001 | <0.001 | 0.001 |
| Max Inverse Radius(mm^-1^) | 0.18(0.17, 0.19) | 0.19(0.17, 0.20) | 0.23(0.21, 0.26) | <0.001 | 0.218 | <0.001 | <0.001 |
| DA Ratio Max[2mm] | 4.04(3.71, 4.36) | 4.61(4.38, 4.84) | 5.94(5.25, 7.01) | <0.001 | <0.001 | <0.001 | <0.001 |
| Pachy Slope(µm) | 44.14(37.37, 51.83) | 45.02(37.81, 48.71) | 66.35(54.64, 82.16) | <0.001 | 1.000 | <0.001 | <0.001 |
| DA Ratio Max[1mm] | 1.56(1.51, 1.61) | 1.61(1.57, 1.65) | 1.72(1.66, 1.81) | <0.001 | 0.006 | <0.001 | <0.001 |
| ARTh | 489.53(429.92, 565.89) | 468.13(410.04, 521.26) | 247.10(167.71, 294.36) | <0.001 | 0.886 | <0.001 | <0.001 |
| bIOP(mmHg) | 16.30(15.40, 18.73) | 15.30(13.98, 16.38) | 14.20(12.45, 15.58) | <0.001 | 0.001 | <0.001 | 0.034 |
| Integrated Radius(mm^-1^) | 8.17(7.56, 8.8) | 9.37(8.83, 9.85) | 12.39(10.77, 14.60) | <0.001 | <0.001 | <0.001 | <0.001 |
| SP A1(mmHg/mm) | 120.06(112.32, 131.52) | 98.73(87.21, 112.29) | 64.27(45.19, 79.72) | <0.001 | <0.001 | <0.001 | <0.001 |
| SSI | 0.97(0.84, 1.16) | 0.85(0.72, 1.00) | 0.65(0.56, 0.77) | <0.001 | 0.076 | <0.001 | <0.001 |
| CBI | 0.00(0.00, 0.02) | 0.06(0.00, 0.34) | 1.00(0.98, 1.00) | <0.001 | 0.033 | <0.001 | <0.001 |
| TBI | 0.04(0.01, 0.24) | 0.36(0.14, 0.88) | 1.00(1.00, 1.00) | <0.001 | 0.001 | <0.001 | <0.001 |
| **Adjusted IOP and corneal thickness** |  |  |  |  |  |  |  |
| Max Inverse Radius(mm^-1^) | 0.20(0.18, 0.21) | 0.19(0.18, 0.20) | 0.22(0.21, 0.23) | <0.001 | 0.372 | 0.001 | <0.001 |
| DA Ratio Max[2mm] | 4.59(4.39, 4.79) | 4.60(4.44, 4.77) | 5.52(5.32, 5.72) | <0.001 | 0.934 | <0.001 | <0.001 |
| Pachy Slope(µm) | 45.25(40.80, 49.69) | 43.56(39.86, 47.26) | 67.64(63.17, 72.10) | <0.001 | 0.565 | <0.001 | <0.001 |
| DA Ratio Max[1mm] | 1.61(1.59, 1.63) | 1.61(1.59, 1.63) | 1.68(1.65, 1.70) | <0.001 | 0.830 | 0.001 | <0.001 |
| ARTh | 467.57(436.73, 498.42) | 470.59(444.88, 496.30) | 294.63(263.63, 325.63) | <0.001 | 0.882 | <0.001 | <0.001 |
| bIOP(mmHg) | 15.40(15.29, 15.50) | 15.55(15.46, 15.63) | 15.47(15.36, 15.57) | 0.085 | 0.054 | 0.417 | 0.266 |
| Integrated Radius(mm^-1^) | 9.41(8.96, 9.86) | 9.32(8.94, 9.70) | 11.53(11.08, 11.99) | <0.001 | 0.763 | <0.001 | <0.001 |
| SP A1(mmHg/mm) | 104.25(101.07, 107.43) | 99.93(97.27, 102.58) | 81.25(78.06, 84.45) | <0.001 | 0.041 | <0.001 | <0.001 |
| SSI | 0.92(0.86, 0.98) | 0.88(0.83, 0.93) | 0.74(0.67, 0.81) | 0.001 | 0.322 | 0.001 | 0.001 |
| CBI | 0.15(0.08, 0.22) | 0.20(0.14, 0.26) | 0.83(0.76, 0.90) | <0.001 | 0.275 | <0.001 | <0.001 |
| TBI | 0.17(0.10, 0.24) | 0.45(0.39, 0.51) | 0.94(0.86, 1.00) | <0.001 | <0.001 | <0.001 | <0.001 |

* Linear mixed effects of three groups, ^#1^ control vs SKC, ^#2^ control vs KC, ^#3^ SKC vs KC

SKC: subclinical keratoconus; KC: keratoconus; IOP: Intraocular pressure; DA ratio: Deformation amplitude ratio; ARTh: Ambrósio’s relational thickness horizontal; bIOP: Biomechanical corrected intraocular pressure; SP A1: Stiffness parameter at the first applanation; SSI: stress-strain index; CBI: Corvis biomechanical index; TBI: Tomographic and Biomechanical Index.

**Supplemental Table 3** Ability of corneal tomographic and biomechanical parameters in identifying pediatric KC eyes

| **Parameters** | **Cut off** | **Specificity** | **Sensitivity** | **Youden index** | **AUC(95%CI)** |
| --- | --- | --- | --- | --- | --- |
| K1 F(D) | >43.8 | 0.923 | 0.692 | 0.615 | 0.894(0.819, 0.946) |
| K2 F(D) | >45.9 | 0.962 | 0.923 | 0.885 | 0.981(0.932, 0.998) |
| Ka(D) | >2.5 | 1.000 | 0.865 | 0.865 | 0.947(0.884, 0.981) |
| Kmean F(D) | >44.8 | 0.942 | 0.865 | 0.808 | 0.961(0.904, 0.989) |
| Kmax F(D) | >47.63 | 1.000 | 0.981 | 0.981 | 0.999(0.964, 1.000) |
| ACT(μm) | ≤522 | 0.904 | 0.923 | 0.827 | 0.946(0.884, 0.981) |
| PCT(μm) | ≤526 | 0.885 | 0.923 | 0.808 | 0.932(0.866, 0.972) |
| TCT(μm) | ≤518 | 0.904 | 0.923 | 0.827 | 0.956(0.897, 0.986) |
| IS-Value | >1.15 | 1.000 | 0.922 | 0.922 | 0.965(0.909, 0.991) |
| FE(μm) | >5 | 1.000 | 0.981 | 0.981 | 0.981(0.932, 0.998) |
| PE(μm) | >12 | 1.000 | 1.000 | 1.000 | 1(0.965, 1.000) |
| BAD-D | >2.48 | 1.000 | 1.000 | 1.000 | 1(0.965, 1.000) |
| IOP(mmHg) | ≤14 | 0.885 | 0.789 | 0.673 | 0.915(0.844, 0.961) |
| Max Inverse Radius(mm^-1^) | >0.199 | 0.904 | 0.827 | 0.731 | 0.916(0.845, 0.961) |
| DA Ratio Max[2mm] | >4.61 | 0.942 | 0.942 | 0.885 | 0.973(0.921, 0.995) |
| Pachy Slope(µm) | >53.79 | 0.827 | 0.808 | 0.635 | 0.864(0.782, 0.923) |
| DA Ratio Max[1mm] | >1.64 | 0.885 | 0.846 | 0.731 | 0.93(0.863, 0.971) |
| ARTh | ≤323.49 | 1.000 | 0.808 | 0.808 | 0.967(0.912, 0.992) |
| bIOP(mmHg) | ≤15.8 | 0.712 | 0.808 | 0.519 | 0.834(0.748, 0.899) |
| Integrated Radius(mm^-1^) | >9.35 | 0.942 | 0.904 | 0.846 | 0.969(0.915, 0.993) |
| SP A1(mmHg/mm) | ≤93.47 | 1.000 | 0.942 | 0.942 | 0.985(0.939, 0.999) |
| SSI | ≤0.78 | 0.846 | 0.781 | 0.627 | 0.879(0.796, 0.938) |
| CBI | >0.48 | 0.981 | 0.942 | 0.923 | 0.99(0.946, 1.000) |
| TBI | >0.51 | 1.000 | 1.000 | 1.000 | 1(0.965, 1.000) |

AUC: area of receiver operating characteristic curve; CI: confidence interval; K1 F: front flat keratometry; K2 F: front steep keratometry, Ka: keratometric astigmatism; Kmax F: the front maximum keratometry; Kmean F: front mean keratometry; ACT: the corneal thickness at the pachy apex; PCT: the corneal thickness at the pupil's center; TCT: corneal thickness at the thinnest point of the cornea; FE: the front thinnest corneal point; PE: the posterior thinnest corneal point; BAD-D: Belin Ambrosio enhanced ectasia total deviation index; IOP: Intraocular pressure; DA ratio: Deformation amplitude ratio; ARTh: Ambrósio’s relational thickness horizontal; bIOP: Biomechanical corrected intraocular pressure; SP A1: Stiffness parameter at the first applanation; SSI: stress-strain index; CBI: Corvis biomechanical index; TBI: Tomographic and Biomechanical Index.

**Supplemental Table 4** Ability of corneal tomographic and biomechanical parameters in identifying pediatric SKC eyes

| **Parameters** | **Cut-off** | **Specificity** | **Sensitivity** | **Youden index** | **AUC(95%CI)** |
| --- | --- | --- | --- | --- | --- |
| K1 F(D) | >41.7 | 0.500 | 0.692 | 0.192 | 0.531(0.431, 0.630) |
| K2 F(D) | >43.1 | 0.539 | 0.635 | 0.173 | 0.537(0.436, 0.635) |
| Ka(D) | ≤1.8 | 0.212 | 0.865 | 0.077 | 0.52(0.420, 0.619) |
| Kmean F(D) | >41.8 | 0.346 | 0.827 | 0.173 | 0.535(0.435, 0.634) |
| Kmax F(D) | >43.53 | 0.500 | 0.673 | 0.173 | 0.547(0.447, 0.645) |
| ACT(μm) | ≤549 | 0.558 | 0.885 | 0.442 | 0.763(0.669, 0.841) |
| PCT(μm) | ≤543 | 0.615 | 0.827 | 0.442 | 0.767(0.674, 0.844) |
| TCT(μm) | ≤549 | 0.519 | 0.923 | 0.442 | 0.777(0.685, 0.853) |
| IS-Value | >0.37 | 0.769 | 0.423 | 0.192 | 0.563(0.462, 0.660) |
| FE(μm) | >1 | 0.385 | 0.769 | 0.154 | 0.618(0.518, 0.712) |
| PE(μm) | >7 | 0.846 | 0.558 | 0.404 | 0.75(0.656, 0.830) |
| BAD-D | >1.14 | 0.827 | 0.673 | 0.500 | 0.817(0.729, 0.886) |
| IOP(mmHg) | ≤15 | 0.789 | 0.654 | 0.442 | 0.779(0.687, 0.855) |
| Max Inverse Radius(mm^-1^) | >0.19 | 0.808 | 0.481 | 0.289 | 0.624(0.523, 0.717) |
| DA Ratio Max[2mm] | >4.37 | 0.769 | 0.769 | 0.539 | 0.81(0.721, 0.880) |
| Pachy Slope(µm) | ≤39.75 | 0.577 | 0.289 | 0.135 | 0.511(0.411, 0.611) |
| DA Ratio Max[1mm] | >1.57 | 0.558 | 0.808 | 0.365 | 0.715(0.618, 0.799) |
| ARTh | ≤546.55 | 0.346 | 0.846 | 0.192 | 0.589(0.489, 0.685) |
| bIOP(mmHg) | ≤15.8 | 0.712 | 0.673 | 0.385 | 0.717(0.620, 0.801) |
| Integrated Radius(mm^-1^) | >8.87 | 0.827 | 0.750 | 0.577 | 0.811(0.722, 0.881) |
| SP A1(mmHg/mm) | ≤108.14 | 0.846 | 0.692 | 0.539 | 0.84(0.755, 0.904) |
| SSI | ≤1.11 | 0.289 | 0.976 | 0.264 | 0.66(0.554, 0.755) |
| CBI | >0.02 | 0.789 | 0.615 | 0.404 | 0.698(0.600, 0.784) |
| TBI | >0.35 | 0.942 | 0.519 | 0.462 | 0.784(0.692, 0.859) |

AUC: area of receiver operating characteristic curve; CI: confidence interval; K1 F: front flat keratometry; K2 F: front steep keratometry, Ka: keratometric astigmatism; Kmax F: the front maximum keratometry; Kmean F: front mean keratometry; ACT: the corneal thickness at the pachy apex; PCT: the corneal thickness at the pupil's center; TCT: corneal thickness at the thinnest point of the cornea; FE: the front thinnest corneal point; PE: the posterior thinnest corneal point; BAD-D: Belin Ambrosio enhanced ectasia total deviation index; IOP: Intraocular pressure; DA ratio: Deformation amplitude ratio; ARTh: Ambrósio’s relational thickness horizontal; bIOP: Biomechanical corrected intraocular pressure; SP A1: Stiffness parameter at the first applanation; SSI: stress-strain index; CBI: Corvis biomechanical index; TBI: Tomographic and Biomechanical Index.

**Supplemental Table 5** Logistic regression for predicting pediatric SKC eye

| **Parameters** | **B** | **SE** | ***P*** | ***OR*** | **95%*CI*** |
| --- | --- | --- | --- | --- | --- |
| **Model 1** |  |  |  |  |  |
| SP A1 | -0.105 | 0.022 | <0.001 | 0.900 | 0.862-0.940 |
| Constant | 11.405 | 2.440 | <0.001 | 89724.735 |  |
| **Model 2** |  |  |  |  |  |
| PE | 0.314 | 0.104 | 0.003 | 1.369 | 1.116-1.679 |
| SP A1 | -0.097 | 0.023 | <0.001 | 0.907 | 0.868-0.949 |
| Constant | 8.590 | 2.592 | 0.001 | 5378.337 |  |
| **Model 3** |  |  |  |  |  |
| PE | 0.400 | 0.122 | 0.001 | 1.491 | 1.173-1.895 |
| DA Ratio Max[2mm] | 1.982 | 0.772 | 0.010 | 7.256 | 1.599-32.941 |
| SP A1 | -0.072 | 0.023 | 0.002 | 0.931 | 0.889-0.975 |
| Constant | -3.245 | 4.821 | 0.501 | 0.039 |  |

SE, stand error; OR, odds ratio; CI: confidence interval; SP A1: Stiffness parameter at the first applanation; PE, Height of the thinnest corneal point on the posterior surface; DA ratio: Deformation amplitude ratio.

**Supplemental Table 6** Delong test results for pairwise comparison of the AUCs in predicting pediatric SKC eye

| **Differences** **Between AUCs** | **CBI** | **TBI** | **Model 1** | **Model 2** | **Model 3** |
| --- | --- | --- | --- | --- | --- |
| BAD | 0.120* | 0.0335 | 0.0226 | 0.0618 | 0.0917* |
| CBI |  | 0.0862 | 0.142* | 0.181* | 0.211* |
| TBI |  |  | 0.0560 | 0.0952* | 0.125* |
| Model 1 |  |  |  | 0.0392 | 0.0692* |
| Model 2 |  |  |  |  | 0.0300 |

AUC: area of receiver operating characteristic curve; CI: confidence interval; BAD-D: Belin Ambrosio enhanced ectasia total deviation index; CBI: Corvis biomechanical index; TBI: Tomographic and Biomechanical Index.

* *P*<0.05

Model 1 y= – 0.105*SP A1+11.405

Model 2 y= 0.314*PE – 0.097*SP A1+8.590

Model 3 y= 0.400*PE +1.982* DA Ratio Max[2mm] – 0.072*SP A1–3.245
